# Preclinical virology profiles of the HIV-1 capsid inhibitors VH4004280 and VH4011499

**DOI:** 10.1128/aac.00309-25

**Published:** 2025-09-03

**Authors:** Chunfu Wang, Haichang Huang, Lourdes Valera, Kyle Parcella, Christiana Iwuagwu, Brian McAuliffe, Paul J. Falk, Donald R. O'Boyle II, Ronald E. Rose, Ricardo Ramírez Padilla, Chunxiang Wu, Yong Xiong, John Kadow, Umesh Hanumegowda, Luca Sardo, Eric P. Gillis, Mark Krystal, Robert A. Fridell

**Affiliations:** 1 Discovery Biology, ViiV Healthcare 326929 , Branford, Connecticut, USA; 2 Discovery Chemistry, ViiV Healthcare 326929 , Branford, Connecticut, USA; 3 Department of Molecular Biophysics and Biochemistry, Yale University 5755, New Haven, Connecticut, USA; IrsiCaixa Institut de Recerca de la Sida, Barcelona, Spain

**Keywords:** antiviral activity, capsid, HIV-1, mechanism of action, qPCR, RAM, resistance-associated mutation

## Abstract

With its high degree of conservation and critical role in multiple steps of the HIV-1 life cycle, the HIV-1 capsid protein presents an attractive therapeutic target. Herein, the virologic properties of the HIV-1 capsid inhibitors VH4004280 (VH-280) and VH4011499 (VH-499), including potency, mechanisms of action, and resistance profiles, are described. VH-280 and VH-499 inhibited panels of HIV-1 laboratory strains and viruses containing capsid sequences from clinical isolates with half-maximal effective concentrations in the picomolar range. Time-of-addition experiments determined that the primary block to HIV-1 replication occurred after nuclear import and before integration; however, measurements of replication intermediates by quantitative polymerase chain reaction, Gag degradation, p24 release, and virion morphology by cryo-electron microscopy indicate that VH-280 and VH-499 also block nuclear import, total reverse transcript production, integration, virion assembly, and maturation. *In vitro* resistance selection identified Q67H, A105E, T107D/N, and combinations of these substitutions as conferring 6- to >5,000-fold reductions in susceptibility to both compounds. Certain resistance-associated mutations selected by other capsid inhibitors also reduced susceptibility. Overall, the preclinical virology profiles and other drug-like properties support the potential of VH-280 and VH-499 as long-acting agents for HIV-1 treatment and prevention.

## INTRODUCTION

The 231-amino acid HIV-1 capsid protein (CA) is the second most conserved protein in the HIV-1 proteome, demonstrating its importance to the HIV-1 replication process (1, 2). Indeed, HIV-1 CA has multiple essential roles in both the early and late stages of the HIV-1 life cycle (3–14). Initially produced as part of the HIV-1 Gag polyprotein, the CA subunit (in conjunction with the adjacent spacer peptide 1 domain) stabilizes Gag–Gag interactions critical to the assembly and budding of new virus particles (3, 4). Mature CA, which is released from Gag after activation of the HIV-1 protease during budding (3), reassembles into ~250 hexamers and exactly 12 pentamers that form an asymmetric fullerene cone structure known as the core (15, 16). The viral core contains the HIV-1 genome and proteins necessary for the early steps of the viral life cycle (17). As such, a key role of mature CA is to shield this ribonucleoprotein complex from host innate immune defenses after viral entry (5, 6). HIV-1 CA also mediates trafficking of the viral replication complex along microtubules to the nuclear pore, where it interfaces with phenylalanine-glycine (FG) domains in the various nucleoporins making up the pore to facilitate translocation of the HIV-1 core across the nuclear envelope (7–11, 18–20). Capsid also interacts with cleavage and polyadenylation specificity factor 6 (CPSF6), which helps complete passage through the nuclear pore and target the replication complex toward specific regions of the host cell genome (11–13, 19, 20).

With key functions in multiple phases of the HIV-1 life cycle and its high degree of amino acid sequence conservation, HIV-1 CA presents an attractive therapeutic target. Efforts to develop CA inhibitors have identified four potential target sites, with the most promising pharmacological target being the pocket located at the interface of neighboring CA molecules that serves as the binding site for nucleoporins and CPSF6 (19–24). PF-3450074 (PF-74) is the prototype CA inhibitor targeting this site (22, 25). Lenacapavir (LEN), which shares a similar binding mode to PF-74, demonstrates picomolar antiviral potency and has a multistage mechanism of action by inhibiting both early and late stages of HIV-1 replication (26, 27). In 2022, LEN was approved for use in heavily treatment-experienced people living with multidrug-resistant HIV-1 (28, 29), thereby establishing a benchmark profile for therapeutic CA inhibitors. In this study, the antiviral activity, mechanisms of action, and resistance profiles of two investigational CA inhibitors that similarly target the CPSF6/nucleoporin binding pocket, VH4004280 (VH-280) and VH4011499 (VH-499), are described. Both VH-280 and VH-499 demonstrated picomolar antiviral activity *in vitro* against a collection of laboratory strains and chimeric viruses containing CA sequences derived from clinical isolates. They potently inhibited both early and late stages of HIV-1 replication and had resistance profiles similar to LEN. Recent reports described the safety profiles and pharmacokinetic properties of these two compounds in people without HIV (30, 31). Together with the preclinical virology profiles reported here, results support the potential of VH-280 and VH-499 as long-acting agents for HIV-1 treatment and prevention.

## RESULTS

### Antiviral activity, cytotoxicity, and human serum shift of VH-280 and VH-499

**Fig 1:**
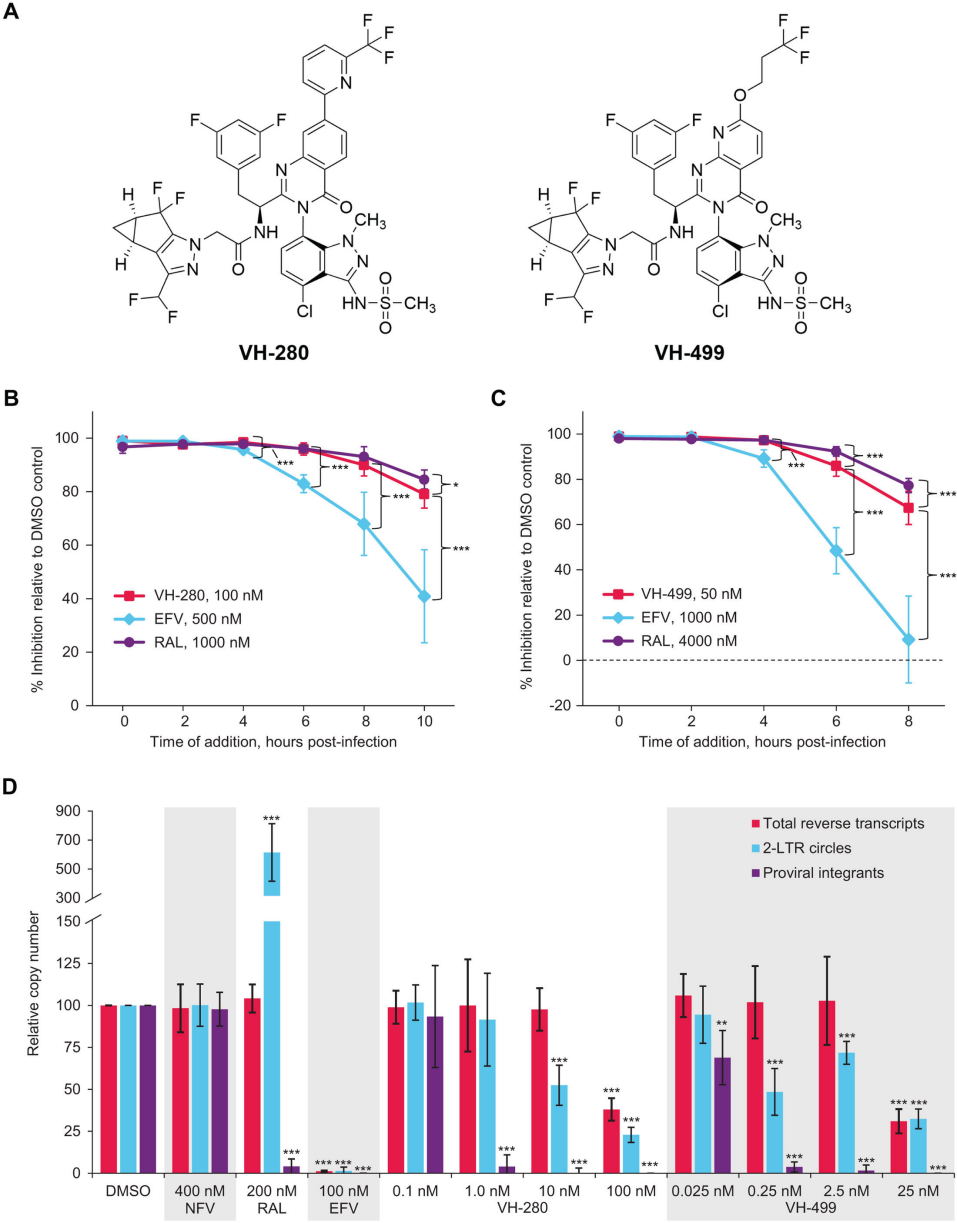
VH-280 and VH-499 (**A**) structures, (**B and C**) time-of-addition experiments, and (**D**) effect on HIV-1 replication intermediates. (**B and C**) VH-280 and VH-499 were added to cultures of MT-2 cells infected with a replication-defective NLRepRlucΔEnv virus pseudotyped with gp160 from the HIV-1 LAI background at the indicated time points. *Renilla* luciferase activity was measured 72 hours post-infection to determine the mean percentage of inhibition relative to DMSO-treated control cells. Time post-infection correlates with progression through the post-entry steps of the virus life cycle; decreases in mean percentage of inhibition are observed if the inhibitor is added after viruses complete the inhibitor-relevant stage(s) of the HIV-1 life cycle. Error bars represent SD. *P* values of *t* test for comparison between the indicated capsid inhibitor and EFV or RAL at each time point. *P < 0.05 and ***P < 0.001. (**D**) MT-2 cells were infected with a replication-defective NLRepRlucΔEnv virus pseudotyped with VSV-G in the presence of the indicated inhibitor. Cells were harvested at 12, 24, and 48 hours post-infection for the quantification of total reverse transcripts, 2 long-terminal repeat circles, and integrated proviruses, respectively. Plotted data represent the number of copies of replication intermediates relative to DMSO-treated control cells. Error bars represent SD from ≥3 experiments. Significance of each *P* value of *t* test was determined relative to DMSO control. **P < 0.01 and ***P < 0.001. DMSO, dimethyl sulfoxide; EFV, efavirenz; LTR, long-terminal repeat; NFV, nelfinavir; RAL, raltegravir; SD, standard deviation; VH-280, VH4004280; and VH-499, VH4011499.

The antiviral activity of VH-280 and VH-499 (Fig. 1A) was evaluated using MT-2 cells and the multi-cycle, replication-competent luciferase reporter virus NLRepRluc-WT, derived from the NL_4-3_ virus (32). As a capsid inhibitor control, LEN was tested in all assays shown in Table 1. Both VH-280 and VH-499 potently inhibited the reporter virus, with mean half-maximal effective concentrations (EC_50_s) of 0.093 ± 0.021 and 0.023 ± 0.008 nM, respectively (Table 1). To confirm the antiviral activity was not due to inhibitor-induced cytotoxicity, the half-maximal cytotoxicity concentration (CC_50_) was assessed in MT-2 cells and found to be >20 µM for both inhibitors. Compound precipitation prohibited evaluation of inhibitor-induced cytotoxicity at higher concentrations. The resulting therapeutic indices (CC_50_/EC_50_) were >220,000 for VH-280 and >870,000 for VH-499. Additionally, the effect of serum protein binding on VH-280 and VH-499 potency was assessed in the presence of 40% human serum (HS), with an additional 27 mg/mL of human serum albumin (HSA) added to achieve an HSA concentration within the normal physiological range (35–50 mg/mL [33]). Under these high serum conditions, the EC_50_ for VH-280 increased to 1.5 ± 0.53 nM, representing a 16-fold increase from standard conditions; for VH-499, the EC_50_ increased by 9.6-fold to 0.22 ± 0.11 nM. The impact of another HS protein, α-1-acid glycoprotein (AGP), on the antiviral activity of VH-280 and VH-499 was evaluated. The data indicated that 1.2 mg/mL of AGP, a concentration within the normal physiological range in human plasma (0.6–1.2 mg/mL [34]), does not affect the potency of VH-280 or VH-499 (VH-280: EC_50_ without AGP, 0.098 ± 0.056 nM [*n* = 4]; EC_50_ with AGP, 0.13 ± 0.069 nM [*n* = 3]; VH-499: EC_50_ without AGP, 0.016 ± 0.008 nM [*n* = 4]; EC_50_ with AGP, 0.017 ± 0.009 nM [*n* = 3]).

**TABLE 1:** Antiviral activity of VH-280 and VH-499 against HIV-1 NLRepRluc-WT and cytotoxicity in MT-2 cells^*a*^

	VH-280	*N*	VH-499	*N*	LEN	*N*
Antiviral activity, standard conditions^*b*^						
EC_50_, mean ± SD (nM)	0.093 ± 0.021	74	0.023 ± 0.008	113	0.018 ± 0.007	219
EC_90_, mean ± SD (nM)	0.23 ± 0.08	74	0.061 ± 0.022	111	0.041 ± 0.015	219
EC_90_/EC_50_ ratio	2.5		2.7		2.3	
Cytotoxicity in MT-2 cells						
CC_50_, mean (µM)	>20	8	>20	4	>20	6
Therapeutic index^*c*^	>220,000		>870,000		>1,100,000	
Antiviral activity, high serum conditions^*d*^						
EC_50_, mean ± SD (nM)	1.5 ± 0.53	38	0.22 ± 0.11	68	0.29 ± 0.13	163
Serum shift factor	16		9.6		16	

^*a*^ CC_50_, half-maximal concentration of cytotoxicity; EC_50_, half-maximal effective concentration; EC_90_, 90% effective concentration; FBS, fetal bovine serum; HS, human serum; HSA, human serum albumin; LEN, lenacapavir; *N*, number of assays, each run in duplicate; SD, standard deviation; VH-280, VH4004280; and VH-499, VH4011499. Data were rounded to two significant digits.

^*b*^ 10% FBS.

^*c*^ Calculated as CC_50_/EC_50_.

^*d*^ 10% FBS + 40% HS + 27 mg/mL of HSA.

### Antiviral activity of VH-280 and VH-499 against laboratory strains and clinical CA sequences

The antiviral activity of VH-280 and VH-499 against additional viral strains was initially evaluated using seven HIV-1 and two HIV-2 laboratory strains in rev-dependent reporter cell lines A3R5-green fluorescence protein (GFP)/Luc and CEM-GFP/Luc, respectively (35, 36). For VH-280, the EC_50_ range was 0.085–0.48 nM, with a median of 0.17 nM, and for VH-499, the EC_50_ range was 0.020–0.069 nM, with a median of 0.042 nM (Table 2). These results were similar to the EC_50_ values obtained in MT-2 cells with the NLRepRluc-WT reporter virus, suggesting that both CA inhibitors are effective against a range of HIV-1 strains, and the different cell backgrounds did not alter inhibitor potency. The two HIV-2 strains examined were also sensitive to both CA inhibitors, although they were less susceptible than the HIV-1 strains (Table 2).

**TABLE 2:** Antiviral activity of VH-280 and VH-499 against HIV-1 and HIV-2 laboratory strains^*a*^

Laboratory strain	Cell line	VH-280		VH-499	
		EC_50_, mean ± SD (nM)	*N*	EC_50_, mean ± SD (nM)	*N*
NL_4-3_	A3R5-GFP/Luc	0.17 (0.13, 0.20)^*b*^	2	0.042 (0.039, 0.046)^*b*^	2
NL_4-3_	CEM-GFP/Luc	0.23 (0.22, 0.23)^*b*^	2	0.036 (0.029, 0.043)^*b*^	2
BAL	A3R5-GFP/Luc	0.13 ± 0.05	4	0.020 ± 0.003	4
HXB2	A3R5-GFP/Luc	0.085 ± 0.045	4	0.026 ± 0.008	4
IIIB	A3R5-GFP/Luc	0.18 ± 0.09	4	0.041 ± 0.008	4
LAI	A3R5-GFP/Luc	0.17 ± 0.08	4	0.043 ± 0.025	4
MN	A3R5-GFP/Luc	0.27 ± 0.10	4	0.054 ± 0.021	4
RF	A3R5-GFP/Luc	0.48 ± 0.17	4	0.069 ± 0.005	4
HIV-1 strains, EC_50_, median (range)^*c*^		0.17 (0.085–0.48)	7	0.042 (0.020–0.069)	7
HIV-2 287	CEM-GFP/Luc	26 ± 8	4	2.1 ± 0.5	4
HIV-2 NIHZ	CEM-GFP/Luc	33 ± 7	4	2.6 ± 0.5	4
HIV-2 strains, EC_50_, median (range)		30 (26–33)	2	2.4 (2.1–2.6)	2


^*a*^ EC_50_, half-maximal effective concentration; SD, standard deviation; VH-280, VH4004280; and VH-499, VH4011499. Data were rounded to two significant digits.

^*b*^ Results from two independent experiments, each run in duplicate.

^*c*^ Data calculated from A3R5-GFP/Luc cell line.

To confirm the ability of VH-280 and VH-499 to inhibit a broad spectrum of HIV-1 isolates, chimeric NLRepRluc viruses containing Gag through protease sequences derived from 48 clinical isolates were evaluated in MT-2 cells. These chimeric viruses were derived from sequences of multiple group M subtypes, including subtypes A, B, C, F, G, and CRF01_AE (37, 38). All chimeric viruses with clinical isolate CA sequences were potently inhibited by both VH-280 and VH-499 (Table 3). Across all viruses, the median EC_50_ was 0.28 nM (range, 0.08–0.93 nM) for VH-280 and 0.058 nM (range, 0.021–0.13 nM) for VH-499. Assay variation was no more than approximately fourfold, as measured by the differences in potency against the integrase strand transfer inhibitor (INSTI) raltegravir (RAL).

**TABLE 3:** Antiviral activity of VH-280 and VH-499 against chimeric viruses with clinical isolate capsid sequences^*a*^

Virus^*b*^	Subtype	VH-280		VH-499		Raltegravir	
		EC_50_, mean ± SD (nM)	*N*	EC_50_, mean ± SD (nM)	*N*	EC_50_, mean ± SD (nM)	*N*
92UG029	A	0.28 ± 0.06	6	0.060 ± 0.009	4	2.5 ± 0.7	8
93TH051	CRF01_AE	0.11 ± 0.03	6	0.025 ± 0.010	4	1.4 ± 0.5	8
93TH062	CRF01_AE	0.16 ± 0.01	6	0.039 ± 0.007	4	2.8 ± 0.7	8
2000166	B	0.08 ± 0.04	4	0.021 ± 0.007	4	1.8 ± 1.0	6
4345	B	0.23 ± 0.01	4	0.029 ± 0.005	4	2.8 ± 0.5	6
91US005	B	0.31 ± 0.05	4	0.070 ± 0.008	4	5.2 ± 2.2	6
91US056	B	0.22 ± 0.05	4	0.040 ± 0.003	4	2.7 ± 0.7	6
92BR003	B	0.28 ± 0.08	4	0.060 ± 0.011	4	3.8 ± 0.7	6
92BR004	B	0.20 ± 0.06	4	0.028 ± 0.003	4	2.7 ± 0.6	6
92BR014	B	0.35 ± 0.26	4	0.068 ± 0.010	4	2.7 ± 1.5	6
92BR020	B	0.19 ± 0.04	4	0.078 ± 0.009	4	5.2 ± 4.7	6
92BR026	B	0.14 ± 0.02	4	0.024 ± 0.007	4	2.3 ± 0.8	6
92BR028	B	0.24 ± 0.13	4	0.088 ± 0.031	8	3.8 ± 2.4	8
92TH026	B	0.12 ± 0.07	4	0.046 ± 0.005	4	2.4 ± 1.6	6
92US660	B	0.16 ± 0.09	4	0.056 ± 0.018	4	2.4 ± 1.2	6
92US712	B	0.24 ± 0.03	4	0.039 ± 0.020	8	2.5 ± 0.8	8
92US714	B	0.22 ± 0.03	4	0.057 ± 0.013	4	3.1 ± 1.3	6
92US715	B	0.13 ± 0.02	4	0.029 ± 0.006	4	2.3 ± 0.8	6
93BR012	B	0.18 ± 0.02	6	0.060 ± 0.010	4	2.9 ± 0.4	8
93BR013	B	0.20 ± 0.04	4	0.041 ± 0.004	4	3.9 ± 0.7	6
93BR015	B	0.22 ± 0.08	4	0.025 ± 0.001	4	2.2 ± 0.7	6
93BR017	B	0.30 ± 0.04	4	0.088 ± 0.005	4	3.1 ± 1.2	6
93BR021	B	0.33 ± 0.11	4	0.058 ± 0.004	4	3.2 ± 1.3	6
93US074	B	0.14 ± 0.05	4	0.049 ± 0.016	4	1.8 ± 1.0	6
93US141	B	0.15 ± 0.05	4	0.039 ± 0.009	4	2.7 ± 0.4	6
93US149	B	0.19 ± 0.01	4	0.030 ± 0.005	4	2.5 ± 1.1	6
ASM57	B	0.15 ± 0.03	4	0.027 ± 0.010	4	2.5 ± 0.9	6
BRH34807	B	0.22 ± 0.01	4	0.046 ± 0.010	4	3.7 ± 2.1	6
USBMS4	B	0.16 ± 0.06	4	0.050 ± 0.007	4	2.2 ± 0.8	6
10215	C	0.43 ± 0.06	4	0.062 ± 0.010	4	1.8 ± 0.5	6
11398	C	0.47 ± 0.13	4	0.079 ± 0.028	4	2.9 ± 1.3	6
20635	C	0.49 ± 0.04	4	0.084 ± 0.013	4	2.3 ± 0.4	6
20706	C	0.47 ± 0.04	4	0.059 ± 0.010	4	2.3 ± 0.8	6
20887	C	0.35 ± 0.07	4	0.060 ± 0.013	4	2.3 ± 0.9	6
21068	C	0.78 ± 0.11	4	0.13 ± 0.03	4	2.8 ± 1.1	6
92IN101	C	0.27 ± 0.06	4	0.064 ± 0.017	4	2.2 ± 0.5	6
92RW026	C	0.35 ± 0.13	4	0.050 ± 0.003	4	2.7 ± 0.5	6
93MW595	C	0.47 ± 0.09	4	0.089 ± 0.034	8	3.8 ± 2.0	8
97ZA003	C	0.43 ± 0.15	4	0.077 ± 0.011	4	3.2 ± 1.3	6
97ZA009	C	0.93 ± 0.22	9	0.090 ± 0.019	10	2.7 ± 1.1	14
98CN006	C	0.38 ± 0.05	4	0.067 ± 0.010	8	3.2 ± 0.7	8
98IN026	C	0.42 ± 0.12	4	0.042 ± 0.019	4	3.1 ± 0.7	6
98TZ017	C	0.33 ± 0.05	4	0.042 ± 0.012	4	2.5 ± 0.5	6
MJ4	C	0.53 ± 0.04	4	0.091 ± 0.006	4	2.7 ± 0.3	6
pC31-8	C	0.50 ± 0.13	6	0.080 ± 0.007	4	2.2 ± 0.4	8
pC40-5	C	0.35 ± 0.07	6	0.066 ± 0.007	4	1.5 ± 0.5	8
93BR020	F	0.43 ± 0.06	6	0.071 ± 0.016	4	3.5 ± 1.1	8
G3	G	0.87 ± 0.34	6	NA^*a*^		NA	
Mean		0.32 ± 0.19	48	0.057 ± 0.023	47	2.8 ± 0.8	47
Median (range)		0.28 (0.08–0.93)		0.058 (0.021–0.13)		2.7 (1.4–5.2)	

^*a*^ EC_50_, half-maximal effective concentration; NA, not applicable; SD, standard deviation; VH-280, VH4004280; and VH-499, VH4011499. Data were rounded to two significant digits.

^*b*^ Chimeric viruses in which the Gag through protease region of the NLRepRluc clone has been replaced with the corresponding region from the indicated HIV-1 clinical isolate.

### Antiviral activity of VH-280 and VH-499 against viruses resistant to other antiretroviral agents

The ability of VH-280 and VH-499 to inhibit viruses with resistance to existing antiretroviral agents was evaluated by assessing their antiviral activity against a panel of antiretroviral-resistant viruses, including a variant with Q67H/N74D substitutions in CA that markedly reduced susceptibility to a previous investigational CA inhibitor (38). As expected, VH-280 and VH-499 inhibited viruses harboring resistance-associated mutations (RAMs) for INSTIs, nucleoside reverse transcriptase inhibitors (NRTIs), non-nucleoside reverse transcriptase inhibitors (NNRTIs), and protease inhibitors (PIs) with approximately equal potency compared with the parent NLRepRluc-WT virus (Table S1). Similarly, no cross-resistance was observed between all other tested antiretroviral agents and the virus containing the Q67H/N74D substitutions in CA. However, increases in EC_50_ of >520- and >1,500-fold were observed for VH-280 and VH-499, respectively, when tested against viruses containing the CA Q67H/N74D substitutions. Altogether, these results demonstrate that VH-280 and VH-499 retain full activity against a variety of antiretroviral-resistant viruses.

### Mechanism of action of VH-280 and VH-499

PF-74, GS-CA1, LEN, and GSK878 inhibit activities associated with both the early (pre-integration) and late (post-integration) stages of the HIV-1 life cycle (25, 26, 38, 39). The potential of VH-280 and VH-499 to inhibit multiple stages of the HIV-1 life cycle was examined. To increase virus infectivity and reduce potential compound carryover in the late assay in a two-cell type system, a highly infectious VSV-G pseudotyped virus was employed. VSV-G pseudotyped virions were generated from HEK 293T cells in the absence (early assay) or presence (late assay) of a CA inhibitor and used to infect an MT-4-derived cell line with integrated copies of an HIV-1 long-terminal repeat (LTR)-driven luciferase reporter gene (B6 cells) (37, 38) in the presence (early assay) or absence (late assay) of a CA inhibitor. The final dilution of the transferred supernatants from HEK 293T cells to B6 reporter cells was 1:2,000, yielding highest possible concentrations of compound carryover in the late assay of 20 pM for VH-280 (40 nM [starting concentration]/2,000 = 20 pM, with 10 threefold serial dilutions of VH-280 on transfected HEK 293T cells) and 5.0 pM for VH-499 (10 nM [starting concentration]/2,000 = 5.0 pM, with 10 threefold serial dilutions of VH-499 on transfected HEK 293T cells). These highest possible concentrations of transferred compounds in the B6 reporter cells were 0.08- and 0.14-fold of the early activity EC_50_ for VH-280 (20 pM/250 pM [early activity EC_50_] = 0.08) and VH-499 (5 pM/36 pM [early activity EC_50_] = 0.14), respectively. Both VH-280 and VH-499 exhibited potent inhibitory activity at both early and late phases (Table 4). More potent mean EC_50_ values were observed for VH-280 and VH-499 when inhibiting the early steps of the life cycle (0.25 and 0.036 nM, respectively) compared with the late steps (2.3 and 0.50 nM, respectively), suggesting their antiviral potency is primarily derived from their effects on the early phase.

**TABLE 4:** Antiviral activity of VH-280 and VH-499 in an early-late assay^*a*^

	VH-280		VH-499		Raltegravir		Nelfinavir	
	EC_50_, mean ± SD (nM)^*b*^	*N*	EC_50_, mean (nM)^*b*^	*N*	EC_50_, mean (nM)^*b*^	*N*	EC_50_, mean (nM)^*b*^	*N*
Antiviral activity^*c*^								
Early activity	0.25 ± 0.04	3	0.036 (0.033, 0.039)	2	4.7 (4.0, 5.3)	2	>300	2
Late activity	2.3 ± 1.3	3	0.50 (0.31, 0.68)	2	>400	3	21 (15, 27)	2
p24 release, IC_50_, mean ± SD (nM)^*b*^	3.9 ± 0.3	3	1.2 (1.1, 1.2)	2	>400	2	150 (150, 150)	2

^*a*^ EC_50_, half-maximal effective concentration; IC_50_, half-maximal inhibitory concentration; SD, standard deviation; VH-280, VH4004280; and VH-499, VH4011499. Data were rounded to two significant digits.

^*b*^ EC_50_ and IC_50_ values are from two or three independent experiments performed in triplicate.

^*c*^ Assay was performed in two different cell lines: HEK 293T cells (virus-producing cells) and B6 reporter cells (MT-4-derived cell line with an integrated HIV-1 LTR-fLuc reporter gene).

To further investigate which early replication step is blocked by VH-280 and VH-499, a time-of-addition assay was performed. MT-2 cells infected with a replication-defective NLRepRlucΔEnv virus pseudotyped with gp160 from HIV-1 LAI were treated with VH-280, VH-499, RAL, or the NNRTI efavirenz (EFV) at specific time points after infection. The inhibitory effect of EFV began to decrease when added 4–6 hours after infection (Fig. 1B through C), indicating reverse transcription had completed in some HIV-1 complexes in the infected cells. In contrast, RAL retained near full antiviral activity when added 4 hours post-infection, only showing a decline when added 6–8 hours after infection. The inhibition profile of VH-280 and VH-499 resembled that of RAL, albeit there was a slightly greater loss of potency compared with RAL at later time points. These results indicate that VH-280 and VH-499 block HIV-1 replication after reverse transcription and slightly before the time of proviral integration.

The mechanism of action of VH-280 and VH-499 was further probed using quantitative polymerase chain reaction (qPCR) assays capable of measuring levels of HIV-1 cDNA (i.e., completion of reverse transcription), levels of 2 long-terminal repeat (2-LTR) circles (indicative of nuclear import), and the presence of integrated proviruses (Fig. 1D) (40). Control inhibitors displayed the expected profiles in the assays, with EFV blocking the production of HIV-1 cDNA and downstream products; RAL having no effect on total reverse transcripts, causing accumulation of 2-LTR circles (40, 41) and blocking integration; and the PI nelfinavir (NFV) having no effect (Fig. 1D). With VH-280 and VH-499, multiple effects were observed. The most pronounced effect caused by VH-280 and VH-499 was the reduction in the number of integrated proviruses at inhibitor concentrations of 1.0 and 0.25 nM, respectively. Both VH-280 and VH-499 also caused a marked reduction in the number of 2-LTR circles, implying that they block nuclear import as well. However, VH-280 required higher concentrations relative to its EC_50_ to achieve results similar to VH-499 (~100× vs ~10×). At the highest concentrations tested (100 nM for VH-280 and 25 nM for VH-499), both CA inhibitors prevented accumulation of reverse transcripts and 2-LTR circles to a similar extent. The similar effect on HIV-1 cDNA production and 2-LTR circle accumulation suggests that the decrease in 2-LTR circles at the highest concentrations was most likely associated with the reduction in reverse transcripts. These results indicate that VH-280 and VH-499 disrupt multiple early HIV-1 replication events and that their antiviral activity is primarily derived from the inhibition of a step occurring after nuclear import and before proviral integration.

Inhibition of post-integration steps could occur by decreasing virion production or rendering released virions non-infectious. To investigate the late inhibitory activity of VH-280 and VH-499, the amount of p24 protein in cell culture medium after transfection of HEK 293T cells was quantified. VH-280 and VH-499 reduced the amount of p24 released relative to dimethyl sulfoxide (DMSO)-treated controls, with half-maximal inhibitory concentrations (IC_50_s) of 3.9 and 1.2 nM, respectively (Table 4). Maximal inhibition was associated with the complete absence of p24 in the culture supernatant, consistent with a quantitative block to virus production. In contrast, NFV, which inhibits maturation but does not block virion production (42), did not substantially reduce the amount of p24-containing protein (mature or immature, mostly in immature form) in culture medium.

**Fig 2:**
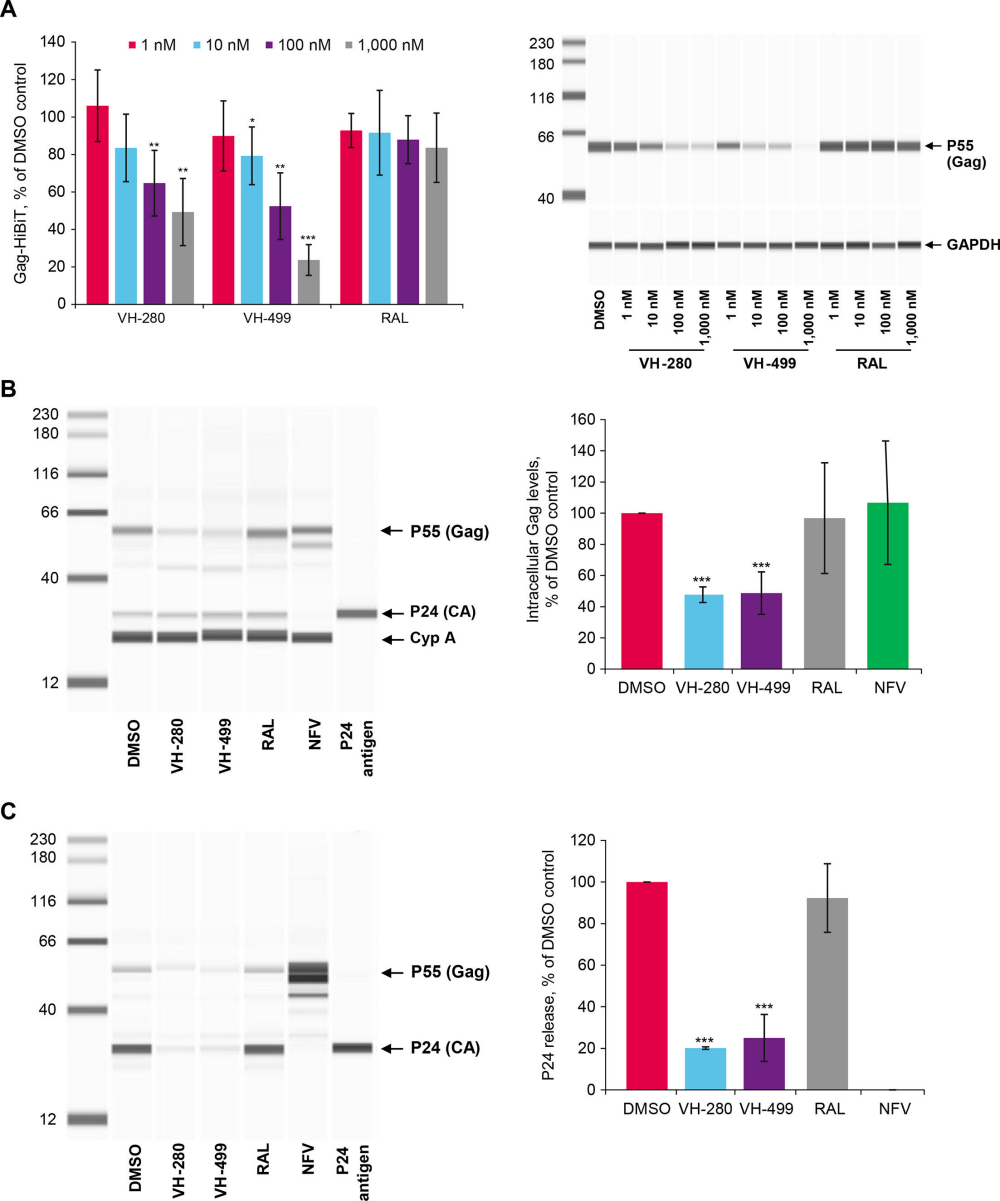
(**A**) Gag degradation in the Gag-THP-1 cell line and reduction in (**B**) intracellular Gag and (**C**) p24 (CA) release in the late stage of the life cycle. (A, left panel) THP-1 cells containing a doxycycline-inducible cassette for the expression of Gag-HiBiT were treated with VH-280, VH-499, or RAL at 1, 10, 100, and 1,000 nM. Luminescence signal was measured by adding HiBiT lytic reagent 24 hours post-treatment. Data were normalized using DMSO control and graphed. Error bars represent SD from four independent experiments. *P* values of the *t* test were calculated in comparison with DMSO control (DMSO set to 100 and not shown). *P < 0.05, **P < 0.01, and ***P < 0.001. (A, right panel) Gag protein from Gag-THP-1 cell lysates was probed using anti-HiBiT monoclonal antibody in an automated Western blot. GAPDH was probed for total cellular protein control using an anti-GAPDH monoclonal antibody. The Western blot image is representative of one of four independent experiments. (**B and C**) A concentration of 300 nM of VH-280 or VH-499 or 1,000 nM of RAL or NFV was added to HEK 293T cells transfected with an NLRepRlucΔEnv virus pseudotyped with gp160 from HIV-1 LAI and cultured for 72 hours. Cells and supernatants were harvested to measure (**B**) intracellular Gag from cell lysates and (**C**) p24 (CA) release from culture medium, respectively, using anti-HIV-1 p55 + p24 + p17 rabbit polyclonal antibodies in an automated Western blot. (**B**) Cyp A was probed for total cellular protein control by anti-cyclophilin A rabbit polyclonal antibody. (**B and C**) Western blot images are representative of one of three independent experiments, with one experiment run in duplicate. The levels of Gag and p24 proteins were quantified by measuring the band area corresponding to Gag or p24, subtracting background signals, and values were normalized to the DMSO control. Error bars represent SD from three independent experiments, with one experiment run in duplicate (total *n* = 4). *P* values of *t* test for comparison between DMSO control and indicated capsid inhibitors. ***P < 0.001. CA, capsid; Cyp A, cyclophilin A; DMSO, dimethyl sulfoxide; GAPDH, glyceraldehyde 3-phosphate dehydrogenase; NFV, nelfinavir; RAL, raltegravir; SD, standard deviation; VH-280, VH4004280; and VH-499, VH4011499.

To determine the cause of the reduction in the amount of p24 in cell culture medium under VH-280 or VH-499 treatment, a series of experiments were performed. These experiments investigated whether VH-280 and VH-499 altered levels of Gag protein in a Gag-expressing THP-1 cell line, intracellular Gag protein levels, and p24 release in HEK 293T cells transfected with virus pseudotyped with the HIV-1 LAI envelope, compared with control antiretrovirals. The THP-1 cell line containing a doxycycline-inducible expression cassette of Gag-HiBiT was treated with VH-280, VH-499, or RAL (control) at concentrations of 1, 10, 100, and 1,000 nM. VH-280 and VH-499 reduced the luminescent signal representing Gag levels to 49% and 24%, respectively, at the highest concentration (1,000 nM; Fig. 2A, left panel), while RAL did not affect Gag protein levels. These data were validated by probing intracellular Gag protein from the same cell lysates using an anti-HiBiT monoclonal antibody in an automated Western blot system (Fig. 2A, right panel). The levels of intracellular Gag and p24 released in the supernatant were measured in HEK 293T cell cultures transfected with virus pseudotyped with the HIV-1 LAI envelope in the presence of VH-280 or VH-499. VH-280 and VH-499 were applied to transfected cells for 72 hours at a concentration of 300 nM, while control compounds (NFV and RAL) were used at 1,000 nM. Intracellular Gag protein from cell lysates was probed using an anti-HIV-1 p55 + p24 + p17 rabbit polyclonal antibody in an automated Western blot system. Data indicated that VH-280 and VH-499 similarly reduced Gag protein levels by ~50% (Fig. 2B, right panel), while NFV and RAL did not affect Gag levels, as expected. In the supernatants obtained from the same samples under VH-280 and VH-499 treatment, p24 protein levels decreased by ~80% and ~75%, respectively (Fig. 2C, right panel). As expected, NFV blocked Gag processing, and no released p24 was detected. RAL did not appear to impact p24 levels. Under the conditions of this experiment, no apparent changes in intracellular p24 were observed (Fig. 2B, left panel). These data suggest that a reduction in intracellular Gag (or Gag degradation) under VH-280 or VH-499 treatment may contribute to the decrease in p24 in cell culture medium, and this change may be associated with the reduction in virion production.

**Fig 3:**
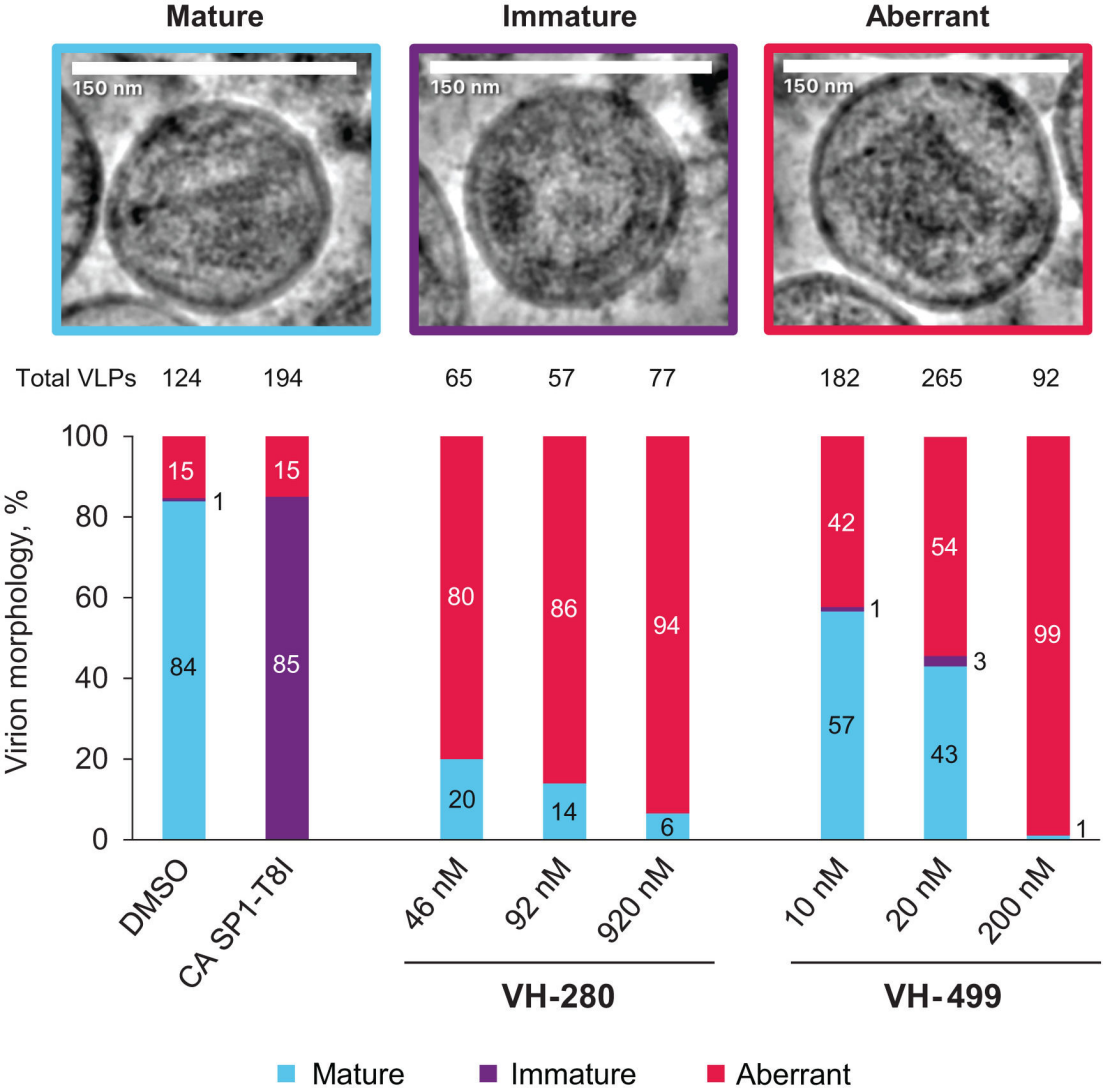
Effect on virion morphology and maturation. Representative images of VLPs treated with VH-499 at 40× late activity EC_50_ (20 nM) are shown at the top. The count of total VLPs is indicated above the graph, and the graph displays the proportions of each morphology. Expi293F cells were treated with VH-280 or VH-499 at 20×, 40×, or 400× late activity EC_50_ (VH-280 46, 92, or 920 nM, respectively; VH-499 10, 20, or 200 nM, respectively) or DMSO, and VLPs were produced via transfection with R9 ΔEnv HIV-1 DNA. Immature CA SP1-T8I VLPs were used as an additional control. Data were collected using a 200 kV Glacios electron microscope and a K3 direct detection camera and analyzed using CryoSPARC software. Images and quantifications were obtained from a single experiment. EC_50_, half-maximal effective concentration; VH-280, VH4004280; VH-499, VH4011499; and VLP, virus-like particle.

The effect of VH-280 and VH-499 on virion morphology and maturation was evaluated using cryo-electron microscopy (Cryo-EM). R9 ΔEnv HIV-1 virus-like particles (VLPs) produced mature VLPs with low amounts of immature VLPs (~1%). In the presence of VH-280 and VH-499 concentrations of 20×, 40×, and 400× late activity EC_50_ (VH-280 46, 92, and 920 nM, respectively; VH-499 10, 20, and 200 nM, respectively), the VLPs exhibited a dose-dependent reduction in the proportion of cone-shaped mature core morphologies compared with DMSO control (Fig. 3). Conversely, the proportion of VLPs exhibiting aberrant core morphologies increased with VH-280 and VH-499 concentrations in a dose-dependent manner. This effect was observed in comparison with the DMSO control and CA SP1-T8I VLPs, which are genetically impaired in their ability to produce mature cores. In the aberrant VLPs, the morphologies appeared to extend from mature lattices since stretches of the polygon-shaped core seemed to be detached from the viral membrane and were not spherical as would be expected for an immature lattice. The aberrant VLPs also exhibited varied sizes of the cores, with some doubling the size of normal mature VLPs. Both VH-280 and VH-499 exhibited potent effects on virion morphology, causing aberrant polygon-like cores of various sizes to form. Of note, these cores displayed stretches of straight lattice that underwent a sharp turn to continue the lattice in another direction. Overall, the increase in virions with aberrant cores under VH-280 and VH-499 treatment may contribute to producing less infectious viruses. This result, in combination with Gag degradation, may explain the late-stage inhibitory activities of VH-280 and VH-499.

Taken together, results from the early-late assay, time-of-addition, qPCR, Gag degradation, p24 release, and virion morphology by Cryo-EM experiments indicate that VH-280 and VH-499 disrupt HIV-1 replication events that take place both pre- and post-integration, including reverse transcription, nuclear import, integration, virion assembly, and maturation. Additionally, the data suggest that the antiviral activity of these compounds is primarily derived from the inhibition of a step occurring after nuclear import but before proviral integration.

### *In vitro* resistance selection toward VH-280 and VH-499

To characterize the resistance profiles of VH-280 and VH-499 *in vitro*, dose-escalating resistance selection studies were conducted in MT-2 cells infected with a cell culture-adapted NL_4-3_ strain carrying a CA H87Q substitution that supports robust replication (43). After three or four passages, sequence analysis of HIV-1 CA amplicons generated from total cellular DNA revealed the emergence of one or more amino acid changes at CA positions 41, 67, and 107 for VH-280 or positions 67, 70, 71, 105, and 107 for VH-499 (Table 5). No changes were observed in amplicons derived from DMSO-treated control cells (data not shown), indicating that the substitutions were selected by the inhibitors. For both VH-280 and VH-499, a Q67H substitution emerged in 100% of the virus population after three and four passages, respectively, and was maintained throughout the remaining passages. At passage 6, 100% of the virus population exposed to VH-280 also included a T107N substitution. This change remained until passage 9, at which point an S41T substitution became fixed in the population, and a mixture of substitutions was detected at position 107. Next-generation sequencing analysis determined that the mixture was composed of ~82% T107D and ~18% T107N. In the cultures treated with VH-499, in addition to the Q67H substitution, an A105E substitution was observed as a minor change at passage 6 but did not become dominant. At passage 7, a complex mixture was observed by population sequence analysis at position 107. Next-generation sequencing determined that 92% of sequence reads were composed of viruses with T107N or T107A at passage 8. At passage 11, viruses with three linked amino acid substitutions predominated, with Q67H/K70R/T107N and Q67H/E71D/T107A being the most prevalent genotypes at ~59% and ~14% of sequence reads, respectively.

**TABLE 5:** Amino acid changes associated with VH-280 and VH-499 treatment*^a^*^,*b*^

Inhibitor	Passage	nM	DPI	CA amino acid change (% of amplicons)*^c^**^,d^*						
				S41	Q67	K70	E71	A105	T107	NGS*^f^*^,^*^g^*
VH-280	P1	0.6	4							
	P2	1.2	7							
	P3	2.4	13		H (100)					
	P4	4.8	15		H (100)					
	P5	9.6	20		H (100)					
	P6	19.2	27		H (100)				N (100)	
	P7	38.4	32		H (100)				N (100)	
	P8	76.8	39	T (90)	H (100)				N (100)	
	P9	153.6	50	T (100)	H (100)				Mixture^*e*^	S41T/Q67H/T107D (82) S41T/Q67H/T107N (18)
VH-499	P1	0.06	4							
	P2	0.12	7							
	P3	0.24	12							
	P4	0.48	20		H (100)					
	P5	0.96	25		H (100)					
	P6	1.92	29		H (90)			E (10)		
	P7	3.84	32		H (100)			E (10)	Mixture	
	P8	7.68	36		H (100)			E (20)	Mixture^*e*^	Q67H/T107A (47) Q67H/T107N (45)
	P9	15.36	42		H (100)				Mixture	
	P10	30.72	46		H (100)	R (60)	D (20)	E (5)	Mixture	
	P11	61.44	49		H (100)	R (60)	D (20)	E (5)	Mixture^*e*^	Q67H/K70R/T107N (59) Q67H/E71D/T107A (14) Q67H/K70R/T107A (6) Q67H/E71D/T107N (5)

^*a*^ CA, capsid; DPI, days post-infection; NGS, next-generation sequencing; PCR, polymerase chain reaction; VH-280, VH4004280; and VH-499, VH4011499.

^*b*^ Parental virus was a tissue culture-adapted version of NL_4-3_ that had an H87Q substitution in CA.

^*c*^ Unless otherwise noted, frequencies were estimated from sequence traces derived from PCR amplicons.

^*d*^ Blank cells (from columns S41 to T107) mean original sequence (WT).

^*e*^ Further analyzed by NGS.

^*f*^ Number of reads for VH-280 passage 9: 28,581; for VH-499 passage 8: 24,789; for VH-499 passage 11: 29,065. Reads < 0.5% of the total were not included in the number of reads used to calculate percentages. Genotypes of reads present at <5% are not shown.

^*g*^ Blank cells in column NGS mean "not performed".

An additional method for selecting viruses with reduced susceptibility to VH-280 or VH-499 was performed. MT-2 cells were infected, treated with fixed concentrations of inhibitor, and monitored for viral breakthrough as indicated by virus-induced cytopathic effect (CPE). Viral breakthrough was observed between Days 20 and 29 at an inhibitor concentration of 3 nM for VH-280 and between Days 7 and 14 at an inhibitor concentration of 0.3 nM for VH-499 (Table S2). Analysis of the proviral DNA isolated from the infected cells treated with VH-280 at viral breakthrough found that Q67H emerged in all six cultures, while no changes in CA amino acid sequence were identified among the infected cells treated with VH-499. Viral breakthrough also occurred on Day 14 in one of six cultures treated with 1.2 nM of VH-499 and was similarly associated with a Q67H substitution. No viral breakthrough was observed after 70 days at inhibitor doses of 12 or 24 nM for VH-280 and 2.4 nM for VH-499.

### Effect of CA inhibitor RAMs on VH-280 and VH-499 antiviral activity

The impact of amino acid changes that emerged during dose-escalating resistance selection experiments on the antiviral activity of VH-280 and VH-499 was examined. Because VH-280 and VH-499 bind to the same pocket as the CA inhibitors PF-74, LEN, and the related CA inhibitor GSK878 (23, 38, 44), the ability of VH-280 and VH-499 to inhibit viruses containing amino acid substitutions associated with reduced susceptibility to other CA inhibitors was also evaluated. Single amino acid substitutions generally resulted in similar fold increases in EC_50_ for VH-280 and VH-499 relative to the wild-type sequence, and these increases ranged from 1.1- to ~6,200-fold (Table 6). Mutations in CA associated with a >3-fold reduced susceptibility to both VH-280 and VH-499 included L56I, M66I, Q67H, Q67Y, N74D, A105E, T107D, and T107N; L56I, M66I, and T107D each caused >400-fold increases in EC_50_. Combinations of CA amino acid substitutions, particularly those that emerged under high selective pressure in the dose-escalating resistance selection studies, also had substantial deleterious effects on VH-280 and VH-499 potency. The double substitutions Q67H/N74D and Q67H/T107D and triple substitutions S41T/Q67H/T107D, S41T/Q67H/T107N, Q67H/K70R/T107N, and Q67H/E71D/T107A caused ≥400-fold changes in EC_50_ to both VH-280 and VH-499. Although neither VH-280 nor VH-499 selected the Q67H/K70R double substitution in resistance selections, this RAM recently emerged in clinical trials studying LEN-containing regimens (45–47). The Q67H/K70R substitution in this RAM analysis was found to reduce virus susceptibility to VH-280 and VH-499 by 160- and 61-fold, respectively (Table 6). A survey of the 11,677 sequences listed in the 2021 Los Alamos National Laboratory database found each of the substitutions or combinations of substitutions that conferred a substantial effect on VH-280 or VH-499 potency to be uncommon (i.e., present in ≤0.06% of sequences) (48).

**TABLE 6:** Effect of capsid inhibitor resistance-associated mutations on VH-280 and VH-499 potency^*a*^

Virus	Prevalence (%)^*b*^	VH-280			VH-499			Raltegravir		
		EC_50_, mean ± SD (nM)	*N*	FC^*c*^	EC_50_, mean ± SD (nM)	*N*	FC^*c*^	EC_50_, mean ± SD (nM)	*N*	FC^*c*^
Wild-type	NA	0.093 ± 0.021	74	1.0	0.023 ± 0.008	113	1.0	2.0 ± 0.8	62	1.0
S41T	34.0	0.25 ± 0.05	12	2.7	0.038 ± 0.011	18	1.7	2.6 ± 0.7	8	1.3
L56I	0.009	71 ± 11	4	760	21 ± 2	6	910	1.9 ± 0.3	8	1.0
M66I	0.034	580 ± 72	4	6,200	38 ± 6	6	1,700	2.0 ± 0.4	10	1.0
Q67H	0.060	0.56 ± 0.13	4	6.0	0.14 ± 0.05	8	6.1	1.6 ± 0.6	6	0.8
Q67Y	0.000	2.7 ± 1.4	4	29	0.43 ± 0.23	3	19	2.0 ± 1.3	6	1.0
K70R	0.043	0.24 ± 0.04	4	2.6	0.040 ± 0.018	8	1.7	2.5 ± 0.9	8	1.3
E71D	45.9	0.19 ± 0.05	8	2.0	0.025 ± 0.005	4	1.1	3.6 ± 1.1	8	1.8
N74D	0.000	2.2 ± 0.4	4	24	0.89 ± 0.21	4	39	2.2 ± 0.4	6	1.1
H87P	1.7	0.21 ± 0.07	70	2.3	0.057 ± 0.019	105	2.5	3.0 ± 1.7	49	1.5
A105E	0.000	5.4 ± 0.7	4	58	1.2 ± 0.1	3	52	2.3 ± 0.9	6	1.2
A105T	0.034	0.27 ± 0.04	8	2.9	0.037 ± 0.005	8	1.6	3.1 ± 0.5	4	1.6
T107A	0.38	0.33 ± 0.11	8	3.5	0.059 ± 0.013	8	2.6	2.2 ± 0.9	10	1.1
T107D	0.000	67 ± 16	12	720	11 ± 2	12	480	3.1 ± 1.0	4	1.6
T107N	0.000	0.67 ± 0.02	4	7.2	0.25 ± 0.03	4	11	2.9 ± 0.7	6	1.5
S41T/Q67H	0.000	3.3 ± 1.1	12	35	0.42 ± 0.12	12	18	2.5 ± 1.2	4	1.3
S41T/T107N	0.000	1.9 ± 0.6	12	20	0.38 ± 0.07	18	17	2.3 ± 0.4	8	1.2
Q67H/K70R	0.000	15 ± 3	8	160	1.4 ± 0.2	8	61	2.1 ± 0.3	5	1.1
Q67H/N74D	0.000	91 ± 30	10	980	43 ± 20	9	1,900	1.7 ± 0.6	16	0.9
Q67H/T107A	0.000	8.2 ± 1.1	8	88	1.8 ± 0.1	8	78	4.0 ± 1.1	8	2.0
Q67H/T107D	0.000	>500	4	>5,400	>170	4	>7,400	2.5 ± 0.6	4	1.3
Q67H/T107N	0.000	9.9 ± 0.2	4	110	3.0 ± 0.5	4	130	3.5 ± 1.2	4	1.8
S41T/Q67H/T107D	0.000	>500	4	>5,400	>500	4	>22,000	2.3 ± 0.5	4	1.2
S41T/Q67H/T107N	0.000	48 ± 6	4	520	9.3 ± 0.4	4	400	2.5 ± 0.5	4	1.3
Q67H/K70R/T107N	0.000	39 ± 3	8	420	14 ± 1	8	610	3.6 ± 1.2	8	1.8
Q67H/E71D/T107A	0.000	64 ± 8	8	690	14 ± 1	8	610	3.3 ± 0.8	8	1.7

^*a*^ EC_50_, half-maximal effective concentration; FC, fold change; SD, standard deviation; VH-280, VH4004280; and VH-499, VH4011499. Data were rounded to two significant digits.

^*b*^ Prevalence (%) among 11,677 Los Alamos National Laboratory database sequences (2021 update).

^*c*^ Ratio of variant virus EC_50_ to wild-type virus EC_50_.

## DISCUSSION

VH-280 and VH-499 are highly potent inhibitors of HIV-1 replication (Table 1), with EC_50_ values against a panel of seven HIV-1 laboratory strains in the range of 85–480 pM and 20–69 pM (Table 2), respectively, and for 47/48 chimeric reporter viruses containing CA sequences from clinical isolates in the range of 80–930 pM and 21–130 pM (Table 3), respectively. Mechanism-of-action studies further determined that VH-280 and VH-499 have multistage inhibitory potential (Table 4; Fig. 1B through D, 2, and 3). Both early and late stages of the HIV-1 life cycle were susceptible to these inhibitors, though the early steps were inhibited ~9.2- to 14-fold more potently than the late steps. As suggested by measurements of proviral integrants and 2-LTR circles, the main block to HIV-1 replication occurred after the import of the viral replication complex into the nucleus and before integration. However, VH-280 and VH-499 were also able to prevent reverse transcription and nuclear import—dependent on drug concentration—as well as virion assembly and maturation (Table 4; Fig. 1D, 2, and 3). *In vitro* resistance selections identified S41T, Q67H, K70R, E71D, A105E, and T107N/D/A as emergent substitutions of interest, though only Q67H, A105E, and T107N/D caused more than fivefold changes in EC_50_ values as individual substitutions (Tables 5 and 6; Table S2). Some substitutions identified as reducing the susceptibility of viruses to LEN, GSK878, and other CA inhibitors (L56I, M66I, Q67Y, N74D, and A105T) also conferred reduced susceptibility to VH-280 and VH-499 to a generally similar extent (26, 38, 39, 49). Importantly, none of the individual substitutions conferring more than fourfold reduced susceptibility were present in >0.06% of Los Alamos National Laboratory database sequences, suggesting that pre-existing resistance to VH-280 or VH-499 should be rare (Table 6).

The timing and location at which the HIV-1 core is disassembled have been recent topics of interest. Older studies suggested it occurred in tandem with cytoplasmic reverse transcription or at the nuclear pore (14, 50, 51), while newer results suggest intact or near-intact cores successfully traverse the nuclear pore complex and do not fully disassemble until after reverse transcription is completed in the nucleus (9, 14, 52–56). The data presented here further support the newer observations. Similar to GS-CA1, LEN, or GSK878 (38, 39, 44), lower concentrations of VH-280 and VH-499 blocked integration without concomitantly decreasing 2-LTR circles, implying that VH-280 and VH-499 primarily inhibit a post-nuclear entry activity. However, the exact near-integration activity that is inhibited remains unknown. Current proposals for how CA inhibitors might achieve post-nuclear block include delaying the release kinetics of viral DNA from the viral core and outcompeting host cofactors for binding to the CA lattice (24). Alternatively, the mechanisms by which LEN, PF-74, and GSK878 disrupt viral replication have been discerned for the higher doses at which nuclear import (38, 44, 55) and/or reverse transcription (24, 38, 44) are also inhibited. Namely, CA inhibitors confer a “lethal hyperstability” to the HIV-1 CA lattice (24). This hyperstability blocks nuclear import by depriving the CA lattice of the elasticity necessary to absorb stresses associated with passage through the nuclear pore complex (55) and stops reverse transcription counterintuitively by causing the viral core to break open (24). Regarding the latter, the binding site for CA inhibitors is adjacent to a motif that modulates CA assembly into pentameric or hexameric multimers (57). The FG motif-containing proteins that act as natural ligands for that binding site preferentially, if not exclusively, bind to hexameric CA multimers (57), suggesting CA inhibitors also favor hexameric CA and may shift the pentamer/hexamer equilibrium further toward hexamers. Consistent with that possibility, PF-74 has been observed to cause the dissolution of pentameric CA structures (22). Therefore, at high enough CA inhibitor concentrations, there may be an insufficient number of CA pentamers to maintain the curvature necessary for a closed (vs tubular) structure (22, 24) and a halt to reverse transcription due to the escape of reverse transcriptase from the viral replication complex (24, 58, 59). VH-280 and VH-499 at high concentrations (VH-280, 100 nM; VH-499, 25 nM) display a mechanism of action similar to that of LEN, PF-74, and GSK878 with regard to inhibition of nuclear import, blocking completion of reverse transcription by stabilizing the CA core, and blocking the CA core opening to release HIV-1 genomic DNA (Fig. 1D). This may explain why the relative copy number of proviral integrants was nearly zero at high concentrations of VH-280 and VH-499 (Fig. 1D).

The detailed mechanism by which CA inhibitors interfere with the late events of the HIV-1 life cycle remains less clear. Since CA inhibitors stop early viral replication by stabilizing the mature CA lattice, disruption of the late stages of HIV-1 replication could have been ascribed to virion maturation inhibition after mature CA monomers are first released. Indeed, virions produced in the presence of LEN or its analog GS-CA1 have misshapen cones suggestive of aberrant CA assembly (26, 39). However, results of the Gag degradation and p24 release assays with VH-280 and VH-499 determined that the block occurred before maturation when virus particles were being assembled, as was also observed for GSK878 and GS-CA1 (38, 39). These results suggest that VH-280 and VH-499 at high concentrations degrade intracellular Gag protein to impair the formation of the immature lattice, presenting a contrast to their known function as stabilizers of the mature lattice. The immature and mature HIV-1 CA lattices are structurally distinct and rely on markedly different CA-CA contacts (60), so these contrasting early and late mechanisms of action are not inherently incompatible. Notably, substituting one of the amino acids within the PF-74 binding site in the amino-terminal region of CA (T107) resulted in a Gag assembly defect and the absence of virus particle production (61). Thus, although the full VH-280 and VH-499 binding sites are likely not present in the immature lattice, extensive contacts between the inhibitors and the amino-terminal region of CA likely remain even in the immature CA lattice, as was also proposed for GSK878. These contacts presumably overlap with a key CA-CA interface necessary for Gag polymerization and consequently inhibit viral assembly. One of the limitations of some mechanism-of-action studies, such as the early/late antiviral activity assay or the quantitative PCR assay to measure HIV-1 replication intermediates, is the use of VSV-G pseudotyped viruses. This approach may result in differences from experiments conducted using viruses with the HIV-1 envelope in terms of viral infection kinetics or other properties of the life cycle. Because a high multiplicity of infection is needed for these assays (e.g., to reduce potential compound carryover in two-cell type systems in the late assay and to improve the detection of integrated HIV-1 DNA), VSV-G or HCMV-G pseudotyped viruses with high infectivity have commonly been used for these types of assays in the field (26, 38, 39, 44, 62).

Cryo-electron microscopy experiments demonstrated that VH-280 and VH-499 treatment induced increased production of aberrant-shaped CA morphologies in VLPs in a dose-dependent manner. This is similar to observations previously reported for VLPs produced by LEN-treated cells (26). Capsid maturation appeared to be affected by the presence of VH-280 and VH-499, resulting in aberrant morphologies stemming from the mature lattice conformation. Low proportions of immature VLPs (0.0%–3.0%) were detected in the samples. This observation differs from a previous study of LEN-treated VLPs (26), but it is consistent with other reports on maturation showing that proportions of immature virions vary (1%–50%) depending on the manual procedures for preparation and determination of morphologies (63, 64).

For both VH-280 and VH-499, Q67H was the only substitution to develop in both of the resistance selection studies, and it reduced the *in vitro* susceptibility of HIV-1 to VH-280 and VH-499 by approximately sixfold. This substitution also emerged during resistance selection with PF-74 and LEN and caused similar low-level impacts on antiviral potency, while minimally affecting viral fitness in tissue culture (26, 65). The only other amino acid position to undergo a major change in both selections was T107 (specifically, T107N/D for VH-280 and T107N/A for VH-499). Notably, Shi and colleagues (65) determined that substitutions at positions Q67 and T107 function as compensatory mutations for other substitutions that have much greater fitness costs (i.e., K70R). Results presented here are consistent with that interpretation, as K70R only emerged after substitutions at Q67 and T107 were dominant. Among the substitutions identified in the resistance selection experiments for VH-280 and VH-499 (Table 5), those that were not previously observed for other CA inhibitors included S41T, E71D, and T107D. However, alternative substitutions have been observed at positions S41 and T107 (49, 65, 66). Position S41 is additionally notable for being outside of the main PF-74 binding pocket (23). Alone, S41T had only a small ≤2.7-fold effect on VH-280 and VH-499 potency. However, the combination of S41T with Q67H and/or T107N resulted in modest (≥17-fold) to high (≥400-fold) decreases in antiviral activity. Notably, S41T is a common polymorphism (~34% of sequences in the Los Alamos National Laboratory database [48]), raising the possibility that pre-existing S41T could potentially lower the barrier to resistance to this inhibitor class. Regarding mutations identified with other CA inhibitors but not with VH-280 and VH-499, a range of sensitivities was observed. The lone substitution located within the cyclophilin A binding loop, H87P, caused a less than threefold change to VH-280 or VH-499 potency, which was consistent with the effect observed for GSK878 and LEN (38). The Q67H/K70R dual substitution has been identified in participants in LEN clinical trials and confers ~50-fold reduced susceptibility to GS-CA1, a close LEN analog, *in vitro* (39, 46, 67). This substitution reduced the potency of VH-280 and VH-499 by ≥61-fold in the present study. The remaining substitutions had more substantial effects, consistent with their impact on other CA inhibitors, but are also known to dramatically reduce viral fitness *in vitro* (68).

In this report, VH-280 and VH-499 were shown to have a multistage mechanism of action, interfering with HIV-1 reverse transcription, nuclear import, integration, virion assembly, and maturation, depending on concentration and time of exposure. Against a panel of viruses with CA sequences from clinical isolates, they were extremely potent inhibitors, preventing infection with EC_50_ values in the picomolar range while also measuring a high therapeutic index. Resistance selections identified six amino acid positions of interest, each located at the contact positions between VH-280 or VH-499 and CA hexamers (69). Substitutions at the majority of these positions are similarly implicated in conferring reduced susceptibility to at least one of PF-74, LEN, GS-CA1, or GSK878 (26, 38, 65, 66, 70). Importantly, none of the substitutions conferring more than fourfold resistance to VH-280 or VH-499 are present in >0.06% of the sequences in the Los Alamos National Laboratory database, suggesting that pre-existing resistance to VH-280 or VH-499 is rare. Additionally, no cross-resistance was observed between VH-280 or VH-499 and other antiretroviral classes (Table S1), establishing their potential for use in combination antiretroviral therapies. Altogether, the preclinical virology profiles of VH-280 and VH-499, as well as their favorable safety and tolerability profiles and pharmacokinetic properties in people without HIV (30, 31), support the potential of VH-280 and VH-499 as long-acting agents for HIV-1 treatment and prevention.

## MATERIALS AND METHODS

### Vectors, viruses, cells, and compounds

VH-280 (95% purity), VH-499 (99.3%–100% purity), LEN (95%–99% purity), RAL (97%–100% purity), EFV (99.7% purity), NFV (100% purity), lamivudine, and atazanavir were prepared by ViiV Healthcare or GSK or purchased from commercial sources. MT-2 cells were obtained from the National Institutes of Health (NIH) AIDS Research and Reference Reagent Program (Bethesda, MD, USA). HEK 293T cells were obtained from American Type Culture Collection (ATCC, Manassas, VA, USA). A3R5-GFP/Luc and CEM-GFP/Luc cells, which contain Rev-dependent GFP and firefly luciferase (fLuc) reporter genes (35, 36), were purchased from 101Bio (Mountain View, CA, USA). The B6 reporter cell line, an MT-4-derived cell line with integrated copies of an HIV-1 LTR-fLuc reporter gene, was obtained from DuPont (Wilmington, DE, USA) (37). The Gag-HiBiT-THP-1 cell line was constructed in-house. Briefly, a doxycycline Gag-HiBiT lentivirus plasmid was constructed using PCR amplification of an NL_4-3_ Gag codon-optimized sequence using primers BRV-347 (5′-GTAGTTTCTAGACGCCGCCACCATGGGCGCCCGC-3′) and BRV-348 (5′-CACACAGAATTCTTAGGATATCTTTTTAAACAGGCGCCATCCACTTACACCTGACGACCCTTGTGACGAGGGGTCGCTGCCAAAGAGT-3′) and cloned into pTMONRB-TRE-MCS-I2-RFP-EFS-rtTA-2A-Blast (Cellecta, Mountain View, CA, USA) as the XbaI and EcoRI fragment. The sequence of the plasmid was confirmed by DNA sequencing. Lentivirus was prepared as previously described (71). THP-1 cells (ATCC) were transduced with the lentivirus, and a stable cell line was selected.

MT-2, B6, and CEM-GFP/Luc cells were propagated in RPMI 1640 medium supplemented with 10% heat-inactivated fetal bovine serum (FBS; GE Healthcare Life Sciences, Marlborough, MA, USA), 10 mM of HEPES buffer (pH 7.5), 2 mM of L-glutamine, 100 units/mL of penicillin G, and 100 µg/mL of streptomycin. A3R5-GFP/Luc cells were propagated in RPMI 1640 medium containing 10% FBS, 100 units/mL of penicillin G, 100 µg/mL of streptomycin, 0.5 mg/mL of G418, and 1 µg/mL of puromycin. HEK 293T cells were propagated in Dulbecco’s Modified Eagle Medium containing 10% FBS, 100 units/mL of penicillin G, and 100 µg/mL of streptomycin. The THP-1 cell line containing a doxycycline-inducible expression cassette for Gag-HiBiT was propagated in RPMI 1640 medium with 10% FBS, Tet system approved (catalog number A4736201, Gibco, Thermo Fisher Scientific, Waltham, MA, USA), 100 units/mL of penicillin G, 100 µg/mL of streptomycin, and 5 µg/mL of blasticidin (catalog number A1113903, Gibco, Thermo Fisher Scientific). Unless otherwise stated, cell culture media and supplements were obtained from Life Technologies (Carlsbad, CA, USA).

Proviral vectors for all HIV-1 and HIV-2 laboratory strains were obtained from the NIH AIDS Reagent Program. The vesicular stomatitis virus G glycoprotein expression vector, pCMV-VSV-G, was purchased from Cell Biolabs (San Diego, CA, USA). pCMV-LAI gp160 was made by inserting gp160-encoding sequences from HIV-1 LAI into a pcDNA expression vector (Thermo Fisher Scientific). The reporter proviral clone, pNLRepRluc-WT, was constructed by transferring the NL_4-3_ coding sequences from pNL_4-3_ to the PvuII site of the pUC18 vector and by replacing the *nef* gene with a reporter gene encoding *Renilla* luciferase (32). A panel of recombinant pNLRepRluc proviral clones with the Gag-Protease (Pr) encoding region derived from HIV-1 clinical isolates has previously been described (37, 38); the clinical isolates were obtained from the NIH AIDS Research and Reference Reagent Program or Bristol Myers Squibb-sponsored clinical trials. pNLRepRluc clones with site-directed mutations in the CA coding region were made with standard recombinant PCR techniques and ligation of restriction enzyme-digested PCR amplicons into the BssHII and ApaI restriction sites of pNLRepRluc-WT or through assembly of the recombinant PCR amplicon with the desired mutation into BssHII and SbfI restriction sites of pNLRepRluc-WT using the NEBuilder HiFi DNA Assembly Master Mix (New England Biolabs, Ipswich, MA, USA). Changes were confirmed by sequence analysis. pNLRepRlucΔEnv was derived from NLRepRluc-WT by introducing a deletion within the *env* gene corresponding to amino acids 133–202. pNLRepRluc clones with site-directed mutations resulting in reduced susceptibility to NRTI, NNRTI, and INSTI antiretrovirals were made with standard molecular biology techniques (72). PI-resistant replicating viruses (Pr M32-pt04 and Pr M37-pt06) were made by replacing Pr-encoding sequences in the NLRepRluc-WT backbone with genes from individuals failing PI treatment with resistance (73).

NLRepRluc virus stocks were generated by transfection of pNLRepRluc vectors into HEK 293T cells using TransIT-293 reagent per the manufacturer’s protocol (Mirus Bio, Madison, WI, USA). Cell culture supernatants were collected ~54 hours post-transfection, clarified by centrifugation (1,000 *g* for 10 minutes), aliquoted, and stored at −80°C. Virus titers were measured in MT-2 cells using luciferase activity as an endpoint for 50% tissue culture infectious dose (TCID_50_) determination (74). Env-pseudotyped virus stocks were likewise generated by co-transfection of pNLRepRlucΔEnv with the appropriate envelope protein expression vector (2:1 ratio of pNL vector to Env expression vector).

### Drug susceptibility and cytotoxicity assays

For the standard HIV-1 multi-cycle (spreading) drug susceptibility assay, MT-2 cells were infected with NLRepRluc virus as previously described (38). Briefly, MT-2 cells were pelleted, resuspended in ~100–300 µL of complete RPMI 1640 medium without phenol red, and mixed with virus by gentle pipetting, followed by incubation at 37°C for ~10 minutes. Additional complete RPMI medium was added, and the mixture was distributed into 96-well assay plates (26,000 cells/well in 200 µL; Corning Life Sciences, Tewksbury, MA, USA) containing threefold serial dilutions of inhibitors in DMSO (2 µL/well) or DMSO alone (2 µL). Background was derived from wells containing complete medium plus DMSO without cells. After incubation at 37°C and 5% CO_2_ for ~96 hours, *Renilla* luciferase activity was measured using EnduRen reagent following the manufacturer’s protocol (Promega, Madison, WI, USA). Plate luminescence was read on an EnVision Multilabel plate reader (PerkinElmer Inc., Waltham, MA, USA). Virus inocula yielding ~100,000–200,000 relative light units per well in DMSO-treated cells were used for the drug susceptibility studies.

The effect of HS on the antiviral activity of VH-280 and VH-499 was assessed using the NLRepRluc-WT/MT-2 cell assay in the presence of medium containing 10% FBS, 40% HS (GeminiBio, West Sacramento, CA, USA), and 27 mg/mL of HSA (Sigma-Aldrich, St. Louis, MO, USA). The effect of AGP (Sigma-Aldrich) on the antiviral activity of VH-280 and VH-499 was assessed in the presence of 1.2 mg/mL of AGP in the standard antiviral assay as described above.

For cytotoxicity assays, uninfected MT-2 cells were plated with serial dilutions of inhibitor, incubated at 37°C and 5% CO_2_ for 4 days, and evaluated for viability with XTT reagent (Alfa Aesar, Ward Hill, MA, USA) as previously described (75).

The sensitivity of full-length HIV-1 and HIV-2 strains to the inhibitors was measured in A3R5-GFP/Luc or CEM-GFP/Luc reporter cells. Assays were performed as described for the NLRepRluc reporter viruses, except assays were performed with 20,000 cells/well; background was obtained from uninfected cells in the presence of 1% DMSO, and luciferase expression was measured with a Bright-Glo luciferase assay system per the manufacturer’s protocol (Promega).

EC_50_, EC_90_, and CC_50_ values were calculated using E-workBook software (ID Business Solutions Ltd, Woking, UK) with four-parameter logistic curve fitting (*Y* = bottom + [top − bottom]/[1 + 10^([logEC^_^50^_^− *X*] × Hillslope)^]). The serum shift factor was calculated by dividing the EC_50_ value obtained in high serum conditions by the EC_50_ value obtained in standard conditions (medium with 10% FBS).

### Assay to determine early vs late antiviral activity

Assays to determine the timing of antiviral activity were performed as previously described (38). Briefly, 4 hours after co-transfection of HEK 293T cells with pNLRepRlucΔEnv and pCMV-VSV-G vectors (at an approximately 2:1 ratio of pNL vector to Env expression vector) using TransIT-293 transfection reagent, cells were transferred to 96-well BioCoat cell culture plates (Thermo Fisher Scientific) in the presence (late assay) or absence (early assay) of serially diluted inhibitors (10 concentrations, threefold dilutions). After 24 hours, culture supernatants were collected, diluted 1:20, and 2 µL was transferred to 96-well plates containing B6 reporter cells in 200 µL of complete medium with (early assay) or without (late assay) serial dilutions of inhibitors. The final dilution of the transferred supernatants from HEK 293T cells to B6 reporter cells was 1:2,000, similar to other published assays to test the late-stage activity of LEN or GS-CA1 (26, 39, 44). After 48–72 hours, luciferase expression was measured using Bright-Glo luciferase reagent. The percentage of inhibition relative to DMSO-treated control cells was used to calculate EC_50_ values with GraphPad Prism software (version 8.0; Dotmatics, Boston, MA, USA). p24 in medium from transfected HEK 293T cells was quantified with an HIV p24 (high sensitivity) AlphaLISA Detection Kit (PerkinElmer Inc.). The concentration of p24 antigen relative to DMSO-treated control cells was used to calculate IC_50_ values with GraphPad Prism software.

### Time-of-addition assay

MT-2 cells were infected with NLRepRlucΔEnv virus pseudotyped with HIV-1 LAI gp160 as previously described (38). After infection, cells were washed and distributed to assay plates, and inhibitors were added at the time of plating and at 2-hour intervals up to 10 hours post-infection. *Renilla* luciferase activity was measured 96 hours post-infection as described above. The percentage of inhibition relative to DMSO-treated control cells was calculated for each time point. A *t* test was used to determine significance (*P* values).

### Resistance selection assays

A cell culture-adapted HIV-1 NL_4-3_ virus was obtained by propagating the full-length NL_4-3_ virus in MT-2 cells for multiple passages and used for resistance selection studies. Aliquots of the virus were stored at −80°C.

For dose-escalating resistance selection assays, MT-2 cells (1 × 10^6^ cells) were infected with cell culture-adapted HIV-1 NL_4-3_ in T-25 cm^2^ cell culture flasks (multiplicity of infection, 0.005). Sixteen hours post-infection, cells were treated with VH-280 (0.6 nM in DMSO), VH-499 (0.06 nM in DMSO), or DMSO (control, 0.2%, vol/vol) and visually monitored every 1–3 days thereafter for virus-induced CPE. In the absence of appreciable CPE, cultures were refreshed every 3–4 days by removing approximately half of the cells and medium and adding an equal volume of fresh growth medium, maintaining the same inhibitor concentration. When CPE was detected in ~80% of cells, the culture medium was collected, and the remaining cells were pelleted and stored at −80°C for genotypic analysis. The culture medium was used to infect fresh MT-2 cells (1:100, vol/vol ratio) with a doubling of the inhibitor concentration. This process of new infection and inhibitor concentration doubling was counted as one viral passage.

For fixed-dose resistance selection assays, MT-2 cells (1 × 10^5^ cells/mL) were infected for 2 hours with cell culture-adapted HIV-1 NL_4-3_ virus (multiplicity of infection, 0.005), pelleted (1,000 *g* for 10 minutes), resuspended in growth medium, and plated in 24-well tissue culture plates (1 mL/well, 2 × 10^5^ cells). At 16 hours post-infection, an additional 1 mL of medium containing VH-280 (final concentration, 3, 12, or 24 nM), VH-499 (0.3, 1.2, or 2.4 nM), or DMSO was added with six replicates per condition. The final DMSO concentration in all wells was 0.2% (vol/vol). Cell cultures were monitored for virus-induced CPE and maintained as described above. In the absence of a viral breakthrough, cell cultures were terminated at 10 weeks post-infection.

### Genotypic analysis

For the genotypic analysis of breakthrough virus in the resistance selection assays, total cellular DNA was isolated from infected cells using a DNeasy Blood and Tissue Kit following the manufacturer’s protocol (Qiagen, Germantown, MD, USA). CA encoding sequences were amplified by PCR (primers: 5′-GATCTCTCGACGCAGGACTCGGCTTGC-3′/5′-CTTTCATTTGGTGTCCTTCCTTTCCAC-3′), and purified amplicons were submitted for population sequence analysis (GENEWIZ, South Plainfield, NJ, USA). Sequence traces were analyzed with Lasergene software (version 15.0; DNASTAR, Madison, WI, USA). Next-generation sequencing was performed at GENEWIZ from a PCR amplicon spanning CA codons 26–129 (primers: 5′-GTAGTAGAAGAGAAGGCTTTCAGC-3′/5′-GATTTCTCCTACTGGGATAGGTGGATTATG-3′).

### Quantitative PCR assays for the detection of replication intermediates

Quantitative PCR assays were performed as previously described (38). MT-2 cells (2 × 10^6^) were infected with DNase-treated NLRepRlucΔEnv virus pseudotyped with VSV-G using the batch pellet infection method. Infected cells were washed three times with complete medium, resuspended, and treated with an inhibitor or DMSO (no inhibitor control, 0.1%, vol/vol). For quantification of total reverse transcripts, 2-LTR circles, and integrated proviruses, total cellular DNA was extracted using a DNeasy Blood and Tissue Kit after 12, 24, or 48 hours of incubation, respectively.

For the analysis of total reverse transcripts and 2-LTR circles, DNA samples were treated with DpnI to remove any methylated DNA left over from vector DNA transfection. Quantitative PCR for total reverse transcripts and 2-LTR circles was performed as previously described (40) with the following modifications: primers and probe for total reverse transcripts were MH531-5′-TGTGTGCCCGTCTGTTGTGT-3′ (forward primer), MH532-5′-GAGTCCTGCGTCGAGAGATC-3′ (reverse primer), and 5′-(FAM)-CAGTGGCGCCCGAACAGGGA-(TAMRA)-3′ (probe) and for the 2-LTR circles, MH535-5′-AACTAGGGAACCCACTGCTTAAG-3′ (forward primer) and MH536-5′-TCCACAGATCAAGGATATCTTGTC-3′ (reverse primer). These were used in combination with a probe spanning the 2-LTR circle junction site: 5′-(FAM)-CTCTAGCAGTACTGGAAGGGCTA-(TAMRA)-3′. Standard curves for the assays were obtained from parallel reactions performed in 12-point in triplicate using 1:4 serial dilutions of control amplicons (4 to 4 × 10^6^ copies) plus a no-DNA control. Quantitative PCR reactions for total reverse transcripts and 2-LTR circles were assembled by mixing DNA (~4% of total cellular DNA for reverse transcripts and ~8% for 2-LTR circles), primers (300 nM), probe (100 nM), and TaqMan Fast Advanced Master Mix (Thermo Fisher Scientific) in 20 µL volumes per the manufacturer’s protocol. Reactions were performed on a QuantStudio 6 Flex System using Real-Time PCR software version 1.3 (Thermo Fisher Scientific). The reaction program consisted of 2 minutes at 50°C, 2 minutes at 95°C, followed by 40 cycles of 95°C for 1 second and 60°C for 20 seconds. All sample values were normalized to GAPDH by the addition of Human GAPDH Endogenous Control Probe (VIC/MGB, primer limited; Thermo Fisher Scientific).

Integrated HIV-1 provirus was measured using a 2-step Alu-PCR method (62). Reactions (50 µL) contained 12.5 ng of sample DNA, 100 nM of Alu forward primer (5′-GCCTCCCAAAGTGCTGGGATTACAG-3′), 600 nM of HIV-1 Gag reverse primer (5′-GCTCTCGCACCCATCTCTCTCC-3′), and 2.5 units of Platinum *Taq* DNA Polymerase (Thermo Fisher Scientific). A second set of reactions was conducted with the HIV-1 Gag primer only (without the Alu-specific primer). An integrated HIV-1 DNA control sample (293T long-term-infected [LTI] HIV-1, a gift from Bushman [76]), was used to derive a standard curve. The first-round PCR program consisted of 95°C for 2 minutes, followed by 20 cycles of 95°C for 15 seconds, 50°C for 15 seconds, and 72°C for 2 minutes and 30 seconds. To convert the LTI HIV-1 integrated DNA reactions to copy number, 10 µL of the first-round PCR was amplified in parallel with serial dilutions of the HIV cDNA total transcript standard, providing a standard curve for the second-round qPCR. Reactions contained 300 nM of HIV-1 LTR forward primer (5′-GCCTCAATAAAGCTTGCCTTGA-3′), 300 nM of HIV-1 LTR reverse primer (5′-TCCACACTGACTAAAAGGGTCTGA-3′), 100 nM of LTR molecular beacon probe (FAM-GCGAGTGCCCGTCTGTTGTGTGACTCTGGTAACTAGCTCGC-Dabcyl), and TaqMan Fast Advanced Master Mix. The second-round qPCR included 10 µL of the first-round PCR samples (including 293T LTI HIV-1 integrated DNA standard), 250 nM of HIV-1 LTR forward primer, 250 nM of HIV-1 LTR reverse primer, 200 nM of LTR molecular beacon probe, and TaqMan Fast Advanced Master Mix. The final integrated provirus copy number for each sample was determined by subtracting the copy number obtained from the HIV Gag primer–only amplification from the Alu forward primer–HIV Gag reverse primer amplification copy number. Copy numbers for total reverse transcripts, 2-LTR circles, and integrated provirus were determined as a percentage relative to the DMSO control. A *t* test was used to determine significance (*P* values).

### Assay to determine intracellular Gag and p24 (CA) release in the late stage of the life cycle

HEK 293T cells (~80% confluent in a 10 cm tissue culture dish) were co-transfected with pNLRepRlucΔEnv and pCMV-LAI-gp160 vectors (at an ~2:1 ratio of pNL vector to Env expression vector) using TransIT-293 reagent following the manufacturer’s protocol. Four hours post-transfection, cells were transferred to 12-well BioCoat cell culture plates at 480,000 cells/well in the presence of a single concentration of inhibitor (VH-280 or VH-499 at 300 nM; control compounds NFV or RAL at 1,000 nM). DMSO was used as a negative control. After 72 hours of treatment, cells and culture medium were harvested for the detection of intracellular Gag and p24 (CA) release, respectively. For intracellular Gag detection, cell lysates were lysed in 100 µL of RIPA buffer (catalog number 89900, Thermo Fisher Scientific) containing 1× protease inhibitor cocktail (catalog number 78430, Thermo Fisher Scientific) after two cold phosphate-buffered saline (PBS) washes. Cell lysates were sonicated to shear genomic DNA and further centrifuged at 12,000 × *g* for 10 minutes at 4°C to remove cell debris. Protein concentration of cell lysates was quantified by Nanodrop 2000 at OD_280_ (Thermo Fisher Scientific). Equal amounts of protein from cell lysates or the same volume of culture medium were loaded and run on the Jess ProteinSimple system (catalog number 004-650, Bio-Techne, Minneapolis, MN, USA) following the manufacturer’s protocol. Anti-HIV-1 p55 + p24 + p17 rabbit polyclonal antibody (1:700 dilution; catalog number ab63917, Abcam, Cambridge, UK) was used for intracellular Gag or p24 (CA) release detection. Anti-cyclophilin A rabbit polyclonal antibody (1:100 dilution; catalog number ab41684, Abcam) was used for cellular control protein cyclophilin A detection. Quantification of intracellular Gag or p24 (CA) release was done using Gag or p24 band area minus background and normalized by the DMSO control using Compass for SW software (version 6.1.0; Bio-Techne). A *t* test was used to determine significance (*P* values).

### Assay to quantitate Gag protein in a Gag-containing cell line

THP-1 cells containing a doxycycline-inducible expression cassette for Gag-HiBiT were counted and diluted in complete RPMI medium to 4 × 10^5^ cells/mL with the addition of 0.25 µg/mL of doxycycline (catalog number D9891, Sigma-Aldrich) for the final concentration. Then, 1 mL/well of cells in medium was added to 24-well tissue culture plates to which 10 µL of DMSO or 10 µL of compounds at 1, 10, 100, and 1,000 nM in DMSO was added. Cells were incubated at 37°C in 5% CO_2_ for 24 hours. After incubation, a 50 µL sample of cells/medium from each well was transferred to a solid white 96-well plate to which a 50 µL/well HiBiT Lytic reagent (catalog number N3040, Promega) was added. The cells/substrate were mixed and read on an EnVision Nexus plate reader (Revvity, Waltham, MA, USA) in luminescence mode. The resulting luminescence signals representing Gag levels were normalized to DMSO control and graphed. A *t* test was used to determine significance (*P* values).

The remaining cells/media were transferred to Eppendorf Tubes (Eppendorf SE, Hamburg, Germany) and spun for 4 minutes at maximum speed in a tabletop centrifuge. Medium was removed by aspiration, and cell pellets were washed in 1 mL of PBS, spun again, and PBS was removed by aspiration. Cell pellets were resuspended in 200 µL of RIPA buffer containing 1× PI (catalog number 78430, Thermo Fisher Scientific) and 1× phosphatase inhibitors (catalog number 78420, Thermo Fisher Scientific) with benzonase (2.5 U/mL; catalog number E8263, Sigma-Aldrich) and incubated on ice for 10 minutes before use. The resulting lysates were used in a Jess ProteinSimple system analysis (4 µL of sample + 1 µL of master mix denaturant) as recommended by the ProteinSimple protocol. Gag protein was probed using anti-HiBiT monoclonal antibody (catalog number N7200, Promega; 1:2,000 in ProteinSimple antibody dilution buffer [042-203]), and cellular protein GAPDH was probed using anti-GAPDH monoclonal antibody (1:50 in antibody dilution buffer; catalog number ab8245, Abcam).

### Cryo-electron microscopy to assess the effect on virion morphology and maturation

#### VLP production

Expi293F cells (Thermo Fisher Scientific) were cultured in suspension shaker flasks (250 rpm) at 37°C in 8% CO_2_. Before transfection, VH-280 or VH-499 in 100% DMSO was added to the cell culture for a final concentration of 20×, 40×, or 400× late activity EC_50_ (VH-280 46, 92, or 920 nM, respectively; VH-499 10, 20, or 200 nM, respectively), with a final DMSO concentration <0.02%. Thirty minutes after inhibitor treatment, VLP production was performed by transfecting 1 µg of R9 ΔEnv HIV-1 DNA (77) per milliliter of culture using the ExpiFectamine 293 Transfection Kit (Thermo Fisher Scientific). Forty-eight hours post-transfection, the culture was centrifuged at 500 × *g* for 5 minutes to pellet the cells, and the VLP-containing supernatant was filtered through a 0.45 µm syringe filter. A 20% sucrose solution in 10 mM of Tris-HCl, 100 mM of NaCl, and 1 mM of EDTA at pH 7.4 (1× STE buffer) was overlaid with the filtered supernatant and centrifuged at 120,000 × *g* for 3 hours at 4°C in an SW 32 rotor (Beckman Coulter, Brea, CA, USA). The resulting pellet was resuspended in 80 µL of 1× STE buffer, aliquoted, and stored at −80°C.

Similarly, immature (78) CA SP1-T8I VLPs were produced as previously described (79). Briefly, Expi293F cells were transfected with 1 µg/mL of codon-optimized Gag expression plasmid (pCMV-Gag-opt) with the immature-stabilizing mutation T8I in the SP1 region while being treated with or without DMSO or CA inhibitor and purified and stored as described above.

#### Cryo-electron microscopy sample preparation and imaging

Capsid inhibitor-treated R9 ΔEnv HIV-1 VLPs were thawed in a 42°C water bath and pelleted at 16,000 × *g* on a tabletop centrifuge for 5 minutes. Supernatant was removed, and the pellet was resuspended in 1× STE buffer and used for subsequent freezing steps. Four microliters of sample was applied to glow-discharged QUANTIFOIL R 2/1 film on 300 copper mesh (Quantifoil Micro Tools GmbH, Großlöbichau, Germany), blotted for 4 seconds, and plunge-frozen in liquid ethane with an FEI Vitrobot Mark IV (Thermo Fisher Scientific). Sample grids were screened, and data were collected using a 200 kV Glacios electron microscope (Thermo Fisher Scientific). Images were collected with a K3 direct detection camera (Gatan, Inc., Pleasanton, CA, USA) at 45,000× magnification, with a physical pixel size of 0.86 Å and a total dose of 50 e/Å^2^, or at 36,000× magnification, with a physical pixel size of 1.12 Å and a total dose of 50 e/Å^2^.

#### Data processing

Cryo-electron microscopy images were analyzed using CryoSPARC (software version 4.4.1; Structura Biotechnology, Inc., Toronto, Canada [80]). Raw movies were motion- and contrast transfer function-corrected using the implementation in CryoSPARC. Micrographs were assessed using a manual picker and low-pass filtered to discern core characteristics. Criteria for classification included roundness of lattice, shape, and integrity of the CA core.

## Data Availability

All relevant data are within the manuscript and its supporting information.

## SUPPLEMENTAL MATERIAL

Supplemental tables aac.00309-25-s0001.pdf Tables S1 and S2.

ASM does not own the copyrights to Supplemental Material that may be linked to, or accessed through, an article. The authors have granted ASM a non-exclusive, world-wide license to publish the Supplemental Material files. Please contact the corresponding author directly for reuse.
