# Supplementary material for: Preclinical virology profiles of the HIV-1 capsid inhibitors VH4004280 and VH4011499
Source: Antimicrob Agents Chemother. 2025 Sep 3;69(10):e00309-25. doi: 10.1128/aac.00309-25 (PMC12486825; doi:10.1128/aac.00309-25)
Supplement: Supplemental tables — Tables S1 and S2. [file aac.00309-25-s0001.pdf]

SUPPLEMENTAL MATERIAL

Table S1. Fold Change in EC_{50} of VH-280 and VH-499 Against NLRepRluc Viruses Containing RAMs to Different Antiretroviral Classes[a]

	CA inhibitor		INSTI	NRTI	NNRTI	PI
**Substitution**	**VH-280**	**VH-499**	**RAL**	**3TC**	**EFV**	**ATV**
CA Q67H/N74D	>520	>1,500	0.9	1.4	1.5	1.1
IN G140S/Q148H	1.1	0.9	460	0.8	0.8	1.0
IN Y143R	1.1	1.0	670	0.9	0.9	0.9
IN N155H	1.2	1.3	15	1.2	2.1	1.2
RT K65R	0.8	0.6	1.3	4.1	0.3	1.4
RT M184V	0.8	0.6	0.9	>7.2	0.8	0.8
RT K103N/Y181C	1.0	1.4	1.1	0.7	19	1.0
PR M32-pt04	1.7	1.2	0.9	1.2	0.3	32
PR M37-pt06	0.9	0.9	1.0	0.5	0.5	45

ATV, atazanavir; CA, capsid; EC_{50}, half-maximal effective concentration; EFV, efavirenz; IN, integrase; INSTI, integrase strand transfer inhibitor; NNRTI, non-nucleoside reverse transcriptase inhibitor; NRTI, nucleoside reverse transcriptase inhibitor; PI, protease inhibitor; PR, protease; RAL, raltegravir; RAM, resistance-associated mutation; RT, reverse transcriptase; 3TC, lamivudine; VH-280, VH4004280; VH-499, VH4011499.

Data were rounded to two significant digits.

[a]Relative to the wild-type NLRepRluc virus (N ≥3).

Table S2. Fixed-Dose VH-280 and VH-499 Resistance Selection[a]

Compound, concentration	Culture day of breakthrough	CA substitution (%)
DMSO	6	None
VH-280		
3.0 nM	20-29	Q67H (100)
12.0 nM	70[b]	None
24.0 nM	70[b]	None
VH-499		
0.3 nM	7-14	None
1.2 nM	14/70[c]	Q67H (100)/None
2.4 nM	70[b]	None

DMSO, dimethyl sulfoxide; VH-280, VH4004280; VH-499, VH4011499.

[a]Each dose examined in six independent cultures. Input virus included CA H87Q.

[b]Experiment discontinued on Day 70 with no viral breakthrough observed. [c]Q67H emerged in only one of six cultures; the remaining five cultures were discontinued on Day 70 with no viral breakthrough observed.
